# Single-cell responses to ionizing radiation

**DOI:** 10.1007/s00411-013-0488-3

**Published:** 2013-08-31

**Authors:** Brian Ponnaiya, Sally A. Amundson, Shanaz A. Ghandhi, Lubomir B. Smilenov, Charles R. Geard, Manuela Buonanno, David J. Brenner

**Affiliations:** Center for Radiological Research, Columbia University, 630 West 168th Street, VC11-240, New York, NY 10032 USA

**Keywords:** Ionizing radiation, Single-cell analyses, qRT-PCR, Endogenous controls

## Abstract

While gene expression studies have proved extremely important in understanding cellular processes, it is becoming more apparent that there may be differences in individual cells that are missed by studying the population as a whole. We have developed a qRT-PCR protocol that allows us to assay multiple gene products in small samples, starting at 100 cells and going down to a single cell, and have used it to study radiation responses at the single-cell level. Since the accuracy of qRT-PCR depends greatly on the choice of “housekeeping” genes used for normalization, initial studies concentrated on determining the optimal panel of such genes. Using an endogenous control array, it was found that for IMR90 cells, common housekeeping genes tend to fall into one of two categories—those that are relatively stably expressed regardless of the number of cells in the sample, e.g., *B2M, PPIA, and GAPDH,* and those that are more variable (again regardless of the size of the population), e.g., *YWHAZ, 18S, TBP, and HPRT1*. Further, expression levels in commonly studied radiation-response genes, such as *ATF3, CDKN1A, GADD45A, and MDM2,* were assayed in 100, 10, and single-cell samples. It is here that the value of single-cell analyses becomes apparent. It was observed that the expression of some genes such as *FGF2 and MDM2* was relatively constant over all irradiated cells, while that of others such as *FAS* was considerably more variable. It was clear that almost all cells respond to ionizing radiation but the individual responses were considerably varied. The analyses of single cells indicate that responses in individual cells are not uniform and suggest that responses observed in populations are not indicative of identical patterns in all cells. This in turn points to the value of single-cell analyses.

## Introduction

It is becoming increasingly clear that cells in a population, even a supposedly homogeneous one, are not identical in terms of gene expression. Studies of single cells, in either resting or stimulated states, have demonstrated large variations among individual cells (Cai et al. 2006; Maheshri and O’Shea 2007; Raj et al. 2006). This complexity has led to the idea that not all cells in a population may behave in exactly the same way in terms of gene expression and that examining the response of a population as a whole can potentially mask subtle differences within the population. This in turn has increased the appreciation for the power of analyzing cellular responses on a cell-by-cell basis (Bengtsson et al. 2008; Marcy et al. 2007; Toriello et al. 2008; Zhang et al. 2008; Zhong et al. 2008).

The analyses of individual cells may prove to be important in the cellular response to ionizing radiation. Radiation is routinely used in studies of DNA repair and cellular responses to DNA damage. Following exposure to ionizing radiation, there is an induction of a host of cellular responses, including stress signaling, cell cycle arrest, and activation of complex DNA repair processes (Lobrich and Jeggo 2007). These responses may occur as a result of alterations in specific protein activities via modifications (Jazayeri et al. 2008; Pilch et al. 2003), changes in subcellular localization (Mladenov et al. 2006; Vissinga et al. 2008), or changes in gene expression profiles (Amundson et al. 2008). While protein modifications and re-localization have been demonstrated in individual cells by immunocytochemistry, to date there is little data on the radiation response in individual cells in terms of gene expression alterations. Almost all studies of gene expression in response to radiation have been based on population studies that have proved invaluable to understanding the complexity of the overall cellular response to ionizing radiation (Amundson 2008).

We have previously reported on the variability of alterations in gene expression in individual irradiated cells (Ponnaiya et al. 2007). A charged particle microbeam was used to target the nucleus of individual cells with a specific number of α-particles. Single cells were isolated using a micromanipulator, and a multiplex RT-PCR protocol was used to amplify *CDKN1A* and *ACTB* products from individual control and irradiated cells. When normalized to *ACTB* expression levels, *CDKN1A* was induced in all irradiated cells at 1-h post-irradiation when compared to controls, but the level of induction varied among individual irradiated cells from four- to ninefold above the mean of the control cells. Obviously, this variation would not be apparent if the population were assayed as a whole.

While the above-mentioned study was designed to demonstrate that it was indeed possible to measure responses to ionizing radiation at the single-cell level, there were several limitations. One of the main constraints was the fact that only a small number of gene products could be assayed reliably from any given cell. In our hands, a maximum of three gene products could be routinely assayed (one of them an endogenous control). This severely limited the power of single-cell analyses to investigate cellular responses to radiation, given the multitude of pathways that may be involved in a cell’s response to irradiation (a few or all of which may be activated in a particular cell). Another weakness to this approach is that conventional RT-PCR is semi-quantitative at best.

To overcome some of the limitations discussed above, we have developed a protocol to increase the number of genes that can be assayed from individual irradiated cells. This approach uses low-density TaqMan real-time PCR arrays that require only a very small amount of material for amplification and quantitative measurement of up to 48 genes in a single cell. TaqMan real-time PCR is an extremely sensitive and reproducible method for detecting gene expression and has been used for single-cell analyses (Citri et al. 2012; Guo et al. 2010; Stahlberg et al. 2013). However, many factors may affect the analysis of the data including the selection of the endogenous control genes. To date there has been little effort to examine the variation of endogenous controls among control and irradiated individual cells. Presumably the best endogenous control would be one that is expressed in all cells at the same level regardless of the experimental conditions. However, experimental evidence suggests that some of the most commonly used control genes (e.g., *GAPDH* and *18S*) are not stably expressed across different cell types or experimental conditions (Glare et al. 2002; Suzuki et al. 2000; Thellin et al. 1999). Therefore, to achieve any accuracy in gene expression analyses, it is important that a careful selection of endogenous controls be conducted. In single-cell analyses, this becomes even more crucial.

In this study, we present a systematic analysis of the stability of expression of a panel of genes routinely used for the normalization of qRT-PCR data using 100, 10, and single-cell samples. In addition, data are presented comparing the responses of 100, 10, and single cells to ionizing radiation as a model for stress response.

## Materials and methods

### Cell culture, irradiation, and single-cell isolation

Low passage IMR90 human lung fibroblasts (Coriell Cell Repository, Camden, NJ) were maintained in a 1:1 mixture of Dulbecco’s Modified Eagle’s medium and Ham’s F10 medium, supplemented with 15 % fetal bovine serum. Prior to each experiment, cells were maintained at confluence for 1 week to ensure that the majority of the population was in G0/G1 phase of the cell cycle. Confluent IMR90 cells were irradiated with 1 Gy γ-rays (0.8 Gy/minute) using a Gammacell-40 ^137^Cs irradiator (AECL, Ontario, Canada) and returned to the incubator for 4 h, following which cells were harvested and resuspended at 1X10^6^ cells/ml in 1 % BSA in PBS. 100, 10, or individual control and irradiated cells were sorted at 4 °C into wells of 96-well plates using a flow cytometer (FACSAria, BD Biosciences). Plates were stored at −20 °C.

### Lysis, reverse transcription, and pre-amplification

Sets of wells were cut from each 96-well plate and the lysis, reverse transcription, and pre-amplification reactions were conducted in the same well to minimize the loss of material. Lysis and RT reactions were performed using the Cells-to-Ct kit (Applied Biosystems, CA). Briefly, wells were thawed on ice and lysed at RT in 1.5 μl of lysis solution. Following lysis, 6.5 μl of RT reaction master mix was added to each well and incubated for 60 min at 37 °C followed by a 5-min incubation at 95 °C. For the single-cell samples (but not the 10 and 100 cell reactions), a pre-amplification reaction was run in the same well using a pre-amplification kit (Applied Biosystems,) using gene-specific probe-primer sets that were identical to those on the low-density arrays. 32 μl of pre-amp master mix was added to each well, and PCR was conducted for 14 cycles according to the manufacturer’s protocol. Following pre-amp, all samples were diluted 1:5 with ddH_2_0 and either loaded onto arrays immediately or temporarily stored at −20 °C.

### Quantitative real-time PCR (qRT-PCR)

qRT-PCRs were performed using Taqman Low-Density Arrays (TLDAs) run on the 7900HT Fast Real-Time PCR System (Applied Biosystems). 50 μl of diluted pre-amp sample was added to 50 μl of Universal PCR master mix (Applied Biosystems), loaded onto the arrays, and run for 45 cycles.

Two types of TLDAs were used in these studies. The first was a preconfigured, Human Endogenous Control TLDA (Applied Biosystems) that has triplicates of 16 genes that are routinely used as normalization controls (*18S, ACTB, B2M, GAPDH, GUSB, HMBS, HPRT1, IPO8, PGK1, POLR2A, PPIA, RPLPO, TBP, TFRC, UBC, and YWHAZ*). The other was a custom-designed array that included 5 endogenous controls (*18S, ACTB, GAPDH, PPIA, and UBC*) and 43 other genes that were selected based on previous observations that they were altered in irradiated and/or bystander populations (Ghandhi et al. 2008).

Baseline and threshold values were automatically determined for all samples using the SDS version 3 software (Applied Biosystems). The obtained data were analyzed using *geNorm,* version 3.5 (Vandesompele et al. 2002), to determine the most stably expressed endogenous control genes. *geNorm* provides a ranking of the tested genes based on the average expression stability value M which is defined as the average pairwise variation of a particular gene compared with all other control genes. Genes with higher M values have greater variations of expression. Additionally, the assessment of the pairwise variations (V_n/n+1_) between each combination of sequential normalization factors allows the identification of the optimal number of reference genes. The geNorm analyses were performed for control and irradiated cells separately as well as by considering all data together. There was no difference in the results as analyzed by either method (data not shown) so the stability of expression of reference genes presented here will focus on the data as a whole with data from both control and irradiated cells combined.

In all the custom array data, the optimal number of reference genes for normalization of the data was determined to be 2. For single-cell analyses, the two genes were *GAPDH* and *UBC,* while for the 10- and 100-cell analyses, the two genes were *PPIA* and *GAPDH*.

## Results

### Comparisons of the stability of expression of endogenous controls

**Fig. 1:**
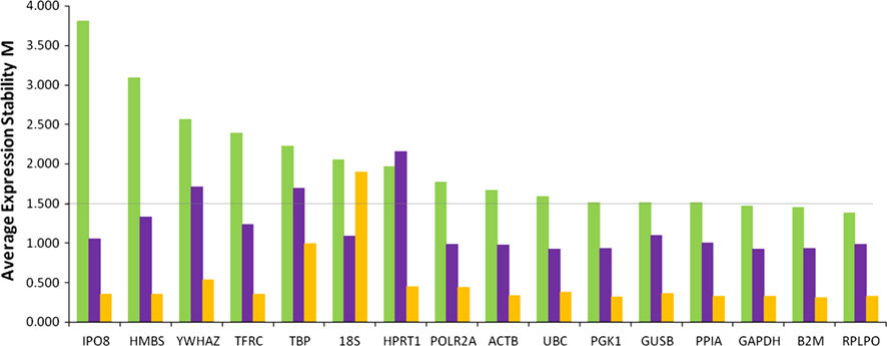
Comparisons of average expression stability (M, as generated by geNorm) of 16 control genes on the endogenous control arrays between samples of single cells (*green bars*), 10 cells (*purple bars*), and 100 cells (*yellow bars*). The *dashed line* indicates an *M* value of 1.5 below which genes are considered to be stable when assayed in populations. Genes are ranked by stability in single cells with the most variable on the *left* and the most stable on the *right*

Since the accuracy of relative quantification by qRT-PCR, especially at the single-cell level, depends greatly on the choice of endogenous control genes that are used for normalization, initial studies concentrated on determining the optimal panel of such genes. The relative expression of 16 housekeeping genes was assayed in control and irradiated samples consisting of 1, 10, or 100 cells (Fig. 1) and analyzed by geNorm (Suzuki et al. 2000).

As can be seen, within the 100-cell analyses, all genes except 18S had *M* values of <1.5 and would be considered stably expressed and appropriate normalization controls for qRT-PCR. When the numbers of cells were reduced to 10, *M* values of three genes (*YWHAZ, TBP,* and *HPRT1*) were above 1.5 which would make them unsuitable endogenous controls. When numbers of cells were reduced to single cells, 10 of the genes rose above a 1.5 M value. Further, with two exceptions (*18S* and *HPRT1*), increasing the number of cells being assayed increased the stability of a particular gene. For example, the most stably expressed gene among single cells, *RPLPO* had an *M* value of 1.387, 0.988, and 0.328 in single-cell, 10-cell, and 100-cell samples, respectively. This is expected that for some genes, there would be more variation across 10 single cells as compared to a pool of 10 cells or 100 cells.

The other trend observed is that, regardless of the number of cells being assayed, some genes such as *YWHAZ*, *18S,* *TBP,* and *HPRT1* tend to be variably expressed, that is, their M values tend to be among the highest for that particular group of cells. Additionally, other genes such as *B2M, PPIA,* and *GAPDH* tended to be among the more stably expressed across all three groups.

### Alterations in the expression levels of genes in response to ionizing radiation

Given the success in observing gene expression at the single-cell level (albeit in relatively highly expressed housekeeping genes) and having determined the optimal number and kinds of endogenous controls required for accurate normalization of the data, we next used custom arrays to determine the expression of radiation-response genes in 100, 10, and individual control and irradiated cells.

**Fig. 2:**
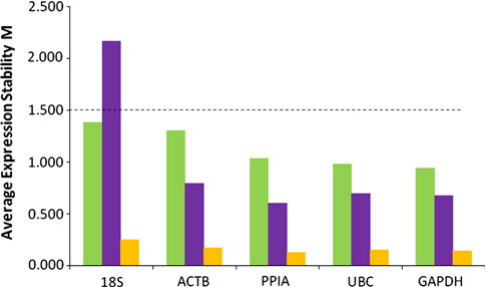
Comparisons of average expression stability (M, as generated by geNorm) of 5 control genes on the custom arrays between samples of single cells (*green bars*), 10 cells (*purple bars*), and 100 cells (*yellow bars*)

Five reference genes were included on the custom arrays, and their average expression stability is presented in Fig. 2. The patterns of expression were similar to those observed in Fig. 1, in which expression of *18S* is most variable while other genes are more uniformly expressed in 100, 10, and single cells. For the single-cell data set, geNorm determined that *UBC* and *GAPDH* were sufficient for normalization of the single-cell data, while *GAPDH* and *PPIA* were most stably expressed in the 10- and 100-cell data and therefore the best reference genes for normalization of these samples.

**Fig. 3:**
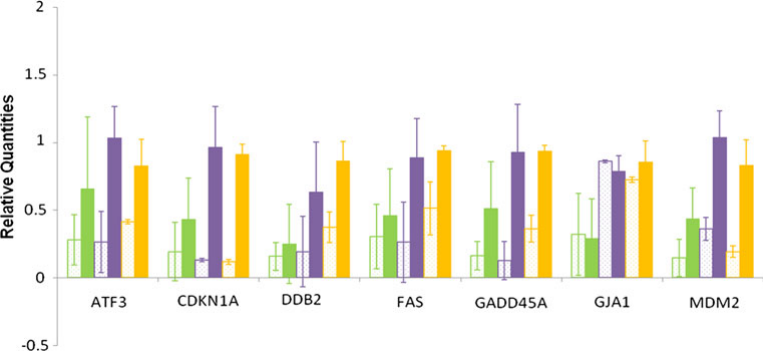
Comparisons of mean relative quantities (±SD) of 7 gene products between non-irradiated (*stippled bars*) and irradiated (*filled bars*) samples of single cells (*green bars*), 10 cells (*purple bars*), and 100 cells (*yellow bars*). All groups were normalized to the most stable pair of endogenous controls within that group: single cells—GAPDH and UBC, 10 and 100 cells—GAPDH and PPIA (see Fig. 2)

Using these endogenous controls, the relative expression levels of 7 radiation-response genes were analyzed in samples of single, 10, and 100 cells (Fig. 3). These genes were consistently detected in all individual cells assayed and allow for the comparison of a gene set across individual irradiated and non-irradiated cells. As can be seen, for 6 of the 7 genes (except *GJA1*), there was good general agreement between the expression profiles observed in individual cells and those seen in 10 and 100 cells samples. When taken together as a group, the expression levels of individual control (non-irradiated) cells were not significantly different from the levels in larger groups of cells with the exception of *GJA1*. Further, similar to patterns seen in 10 and 100 cells irradiated samples, irradiated single cells on average showed higher levels of expression when compared to controls. Interestingly, for *GJA1* while there was a difference in the expression levels between single and 10 and 100 cell samples, there was no induction following exposure to radiation in any of the irradiated populations when compared to the matched controls. Also, as expected, increasing the number of cells per sample, going from 1 to 10 to 100 cells, resulted in smaller variation within the samples (as seen by reduced standard deviations).

**Fig. 4:**
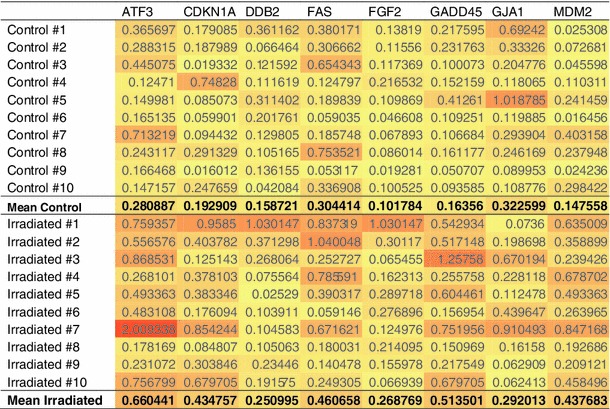
Heatmap depicting relative expression levels of the 7 genes described in Fig. 3 (columns) in 10 individual control and 10 irradiated cells (*rows*). *Colors* represent relative levels of expression with *yellow* being low and *red* being high

While comparing the means of the control group and irradiated group of individual cells demonstrates that the irradiated group had elevated levels of expression of almost all genes, a more detailed examination of the response of individual cells reveals a more complex picture (Fig. 4). Looking at the expression profiles in the individual control cells, it is clear that there is some variability in the basal expression levels of all the genes studied. While some of them, e.g., *FGF2* and *MDM2,* had relatively small degrees of variability, others such as *GJA1* and *FAS* had larger ranges of expression. Irradiated individual cells also demonstrated a range of expression levels over all the genes. Additionally, not all irradiated cells had the same expression profiles. For example, while irradiated cell #1 had elevated expression levels for all genes except *GJA1*, irradiated cell #8 had gene product levels similar to the mean of all control cells. Further, irradiated cell #7 had some of the highest levels of *ATF3, CDKN1A,* and *MDM2*, but control levels of *DDB2* and *FGF2*. On the other hand, irradiated cell #3 had some of the highest levels of *ATF3, GADD45,* and *GJA1* but not for the other genes. Taking the data of 10 irradiated cells for this limited number of genes, it appears that almost all cells respond to ionizing radiation but the pattern of the individual responses is markedly different. Additionally, there is no situation where the expression profile of multiple cells is exactly the same and the response of the population is more heterogeneous at the single-cell level.

## Discussion

Much of what is known of the alterations in gene expression profiles has come from the data that measure expression in RNA pools from thousands, if not millions, of cells. However, there is an awareness of the cell-to-cell variations within a population and the power of single-cell analyses to study this heterogeneity and to focus on effects in rare cells of interest, such as stem cells.

One question that arises in any single-cell analysis is that of the source of variability among individual cells. Essentially, the variations can be due to two reasons—either they reflect true cell-to-cell differences in gene expression or they arise from noise associated with measurements of extremely small amounts of material (femtograms). There has been a significant effort made to examine the relative contributions of each to single-cell analyses. For example, Bengtsson et al. (2008) concluded that the noise in single-cell qRT-PCR is insignificant compared to biological cell-to-cell variations in mRNA levels for medium- and high-abundance transcripts. We believe that in the data presented here, the variations are real differences between cells. First, the mean of the individual irradiated cells was higher than the mean of the control cells for all genes that have been previously shown to be elevated in irradiated populations. Further, these means (of both irradiated and control cells) were in agreement with the data trends for 10 and 100 cell samples. From Fig. 4, it can be seen that there are larger variations among the irradiated cells than within controls. If the genes analyzed are being induced in irradiated cells (as would be expected), the noise would be expected to decrease due to the increase in the number of transcripts. This would indicate that the variations we observe are indicative of cell-to-cell differences and that the contributions of technical noise to these variations are minimal.

The preceding discussion underscores the absolute requirement of a thorough analysis of the stability of endogenous genes used to normalize qRT-PCR data. From our data, it is clear that some genes that are routinely used to normalize RT-PCR data, such as *18S, HMBS,* and *HPRT1,* were very unevenly expressed in our cell system. Additionally, exposure to ionizing radiation did not change the expression levels of the housekeeping genes assayed to any great extent (data not shown). Some genes were always among the most variable irrespective of whether they were assayed at the level of a single cell or in larger populations. Others were stable in 1, 10, or 100 control or irradiated cells. This consistency in the relative stability regardless of cell numbers would suggest that the differences among individual cells are truly biological in nature and not due to some experimentally induced errors. It must be stressed that while the single-cell data are in good agreement with those from 10- and 100-cell samples, care must be taken in the interpretation of the data given the limited number of individual cells.

Radiation-induced transcriptional profiles of fibroblast cells have been analyzed by several groups by microarrays and qRT-PCR (Ding et al. 2005; Iwakawa et al. 2008; Kis et al. 2006; Sokolov et al. 2006; Sugihara et al. 2008; Zhou et al. 2006). However, with exceptions, small changes in gene expression (less than twofold) are typically not considered significant and remain unreported. One exception is Sokolov et al. (2006), who found that in IMR-90 cells irradiated with 1 Gy γ-rays, changes in the level of the majority of the genes assayed were less than twofold. Interestingly, at 2-h post-irradiation, there was about a 2.5-, 1.5-, and 1.4-fold induction in *CDKN1A, GADD45A,* and *DDB2,* respectively, by RT-PCR. Ding et al. (2005) reported that in normal human skin, fibroblasts irradiated with 4 Gy X-rays and assayed by RT-PCR, *MDM2* was 2.2 times higher than controls at 2 h, and there was a 6.5-fold induction of *CDKN1A* 4-h post-irradiation. Further, Kis et al. (2006) irradiated primary human fibroblasts, established from skin biopsies, with 1 Gy γ-rays and reported about a fivefold induction of *CDKN1A* and ~1.5-fold induction in *GADD45A*, 2-h post-irradiation. Our data from single-cell analyses are consistent with these studies, indicating changes in gene expression in individual cells within the range of changes previously reported in studies that employed similar cell types, similar types and doses of radiation, and the same assay method within the same time frame post-irradiation.

What is not apparent in the above-mentioned studies is the heterogeneous nature of the response within the irradiated population. From the data in Fig. 4, it is clear that there is considerable variation both in individual control cells as well as in the responses of irradiated single cells and that this variation is not simply that some cells respond and some cells do not respond. It appears that within a population, the overall response is more complex, with responding genes within a cell being differentially expressed, possibly due to differences between cells in the timing of response between individual cells. Further, expression levels for any particular gene may be relatively uniformly elevated among all cells (e.g. *MDM2*), in which case the data of the population as a whole is very similar to that of most of the individual cells analyzed. Alternatively, a gene may not be expressed uniformly across the population (e.g., *FAS*), in which case the elevation in the expression of the gene seen in the population as a whole is due to elevations in only a fraction of the cells in a population. This would suggest that responses are not uniform across all cells and that the overall response of a population is the sum of a complex pattern of alterations in the expression of a variety of genes in individual cells. These differences would be impossible to detect in analyses of populations.

The variations in responses observed across individual irradiated cells suggest that there may be differences between cells that are inherent in even a predominantly G0 population. It is possible that there is some sort of genetic heterogeneity in the population that results in differences in the way cells respond to damage following irradiation. Alternatively, while the experiments were designed to minimize the effects of cell cycle-related differences, it remains possible that some of the variability observed is related to cells being at subtly different points of the cell cycle.

The data presented here speak to the sensitivity of qRT-PCR to detect small changes in gene expression profiles and together with single-cell analyses provide a powerful approach to investigate the complexity of cellular responses to ionizing radiation, and many other applications where gene expression patterns in individual cells may be of interest.
